# Hippocampal Volume as a Putative Marker of Resilience or Compensation to Minor Depressive Symptoms in a Nonclinical Sample

**DOI:** 10.3389/fpsyt.2019.00467

**Published:** 2019-07-12

**Authors:** Bianca Besteher, Letizia Squarcina, Robert Spalthoff, Marcella Bellani, Christian Gaser, Paolo Brambilla, Igor Nenadić

**Affiliations:** ^1^Department of Psychiatry and Psychotherapy, Jena University Hospital, Jena, Germany; ^2^Fondazione IRCCS Ca’ Granda Ospedale Maggiore Policlinico, University of Milan, Milan, Italy; ^3^Section of Psychiatry, Department of Neurosciences, Biomedicine and Movement Sciences, University of Verona, Verona, Italy; ^4^Department of Neurology, Jena University Hospital, Jena, Germany; ^5^Department of Neurosciences and Mental Health, Fondazione IRCCS Ca’ Granda Ospedale Maggiore Policlinico, University of Milan, Milan, Italy; ^6^Department of Pathophysiology and Transplantation, University of Milan, Milan, Italy; ^7^Department of Psychiatry and Psychotherapy, Philipps Universität Marburg, Marburg, Germany; ^8^Marburg University Hospital—UKGM, Marburg, Germany

**Keywords:** depression, imaging, neuroanatomy, healthy subjects, gyrification, surface-based morphometry, subclinical, voxel-based morphometry

## Abstract

Case-control studies in major depression have established patterns of regional gray matter loss, including the hippocampus, which might show state-related effects dependent on disease stage. However, there is still limited knowledge on compensation effects that might occur in people resilient to depression showing only subclinical symptoms. We used voxel-based morphometry on a multicenter data set of 409 healthy nonclinical subjects to test the hypothesis that local hippocampal volume would be inversely correlated with subclinical depressive symptoms [Symptom Checklist 90-Revised (SCL-90-R) depression scores]. Our region-of-interest results show a significant (*p* = 0.042, FWE cluster-level corrected) positive correlation of SCL-90-R scores for depression and a left hippocampus cluster. Additionally, we provide an exploratory finding of gyrification, a surface-based morphometric marker, correlating with a right postcentral gyrus cluster [*p* = 0.031, family-wise error (FWE) cluster-level corrected]. Our findings provide first preliminary evidence of an inverse relationship for subjects in the absence of clinical depression and might thus point to processes related to compensation. Similar effects have been observed in remission from major depression and thus deserve further study to evaluate hippocampal volume not only as a state-dependent marker of disease but also of resilience.

## Introduction

Major depression is associated with brain structural changes documented in several imaging studies. A recent mega-analysis has demonstrated brain matter reductions in multiple cortical areas, including the orbital frontal cortex, cingulate and insular cortex, and temporal lobes (1). Many of these changes are already evident at first episode (2). Of these changes, the hippocampus is of particular interest because it has been proposed as both a state marker and a putative biomarker for treatment outcome in major depression (3). Hippocampal volume has repeatedly been suggested as a predictor of treatment, including pharmacological antidepressive treatment (4) and electroconvulsive therapy (5, 6). Hippocampal volume has also been shown to potentially distinguish between remitting and nonremitting patients at 1-year follow-up (7). Although there might be some genetic effects of liability to depression related to hippocampal volume (8), there are clearly state-dependent effects related to depressive phenotype in hippocampal gray matter volume (9).

Resilience to psychopathology and compensation are underestimated factors in these anatomical models of depressive symptoms and depressive disorder. These might not be captured in typical approaches to imaging studies of depression, which rely on case–control designs. Although these studies have largely increased our understanding of the anatomy of major depression, they still lack good mechanistic models for compensation that leads to either remission of existing symptoms or individual resilience preventing individuals from developing manifest depressive psychopathology. Also, they do not account very well for dimensional approaches to depression. Not only do depressive symptoms occur in a number of psychiatric conditions other than major depression, they might also arise (at least transiently) in a large proportion of nonclinical subjects, for example, after stressful life events or at times of higher stress. Many individuals suffering such stress, often related to environmental impact, however do not develop manifest depression. Also, depressive symptoms are very common in a number of other psychiatric disorders, such as anxiety disorders, somatization, eating disorders, psychosis, and so on. Therefore, our understanding of hippocampal structure and a dimensional depressive phenotype will have to depend on studying subjects across a wider spectrum of both clinical pathologies and the subclinical spectrum (i.e., subjects without a psychiatric disorder).

Indeed, in recent studies, healthy subjects with a family history of major depression who show resilience to developing a clinical phenotype have been shown to have relatively greater or intact hippocampal volume (10). If relatively larger or preserved hippocampal volume is predictive of a higher probability for remission in major depression and higher hippocampal volume in nonclinical subjects might be a potential indicator of resilience, one would expect to find not only negative correlations between regional brain volumes and depressive symptoms, but also positive correlations indicative of resilience and overcompensation. In a recent study of 177 healthy participants (11), we identified positive gray matter correlations with depressive, anxiety, and somatization symptoms across the number of cortical regions. Several smaller studies in healthy subjects have identified either similar or nonoverlapping cortical areas showing both negative and positive correlations to minor depressive symptoms.

In the present study, we sought to use a large data set from a multicenter study (12, 13) to test specifically the hypothesis that hippocampal volume (as well as other regional brain volumes) might also show anticorrelation with minor nonclinical symptoms of depression. Testing this hypothesis in a nonclinical data set would allow us to avoid potential contamination induced by either changes related to the manifest emergence of a psychiatric disorder or effects related to medication or other forms of treatment. We therefore tested this hypothesis first for the hippocampus and then in addition on a whole-brain level to identify in particular those areas showing positive correlations to nonclinical depressive symptomatology as a potential indicator of resilience.

## Methods

### Participants

For this study, we relied on a multicenter sample of 409 clinically healthy young adults from Jena, Germany, and Verona, Italy, for which we have already published analyses on aggressive traits (12) and subclinical agoraphobic symptoms (13). The sample also includes a subsample of 177 healthy subjects, for whom we have published voxel-based morphometry (VBM) analyses of subclinical depressive, general anxiety, and somatization symptoms (11). As outlined in our previous work on this multicenter sample, this included three subsamples: 177 healthy subjects from Jena (Jena-1 sample), 141 healthy subjects from Jena (Jena-2 sample) for whom data were acquired after a major hardware and software upgrade of the magnetic resonance imaging (MRI) scanner and who are thus treated as a separate sample, and finally 91 healthy subjects from Verona, Italy (Verona/Milano sample).

All study participants provided written informed consent to protocols approved by the local Ethics Committee of Jena University Medical School and the Ethics Committee of the Azienda Ospedaliera Universitaria of Verona.

Demographic details and recruitment for this multicenter cohort have been described previously (12, 13). In brief, we included healthy subjects meeting the following criteria: age 18 or above and ability to provide written informed consent. The exclusion criteria defined for both samples were current or previous psychiatric disorders (axis I), central nervous neurological disorders, traumatic brain injury with loss of consciousness, learning disability, or contraindication for MRI scans, major neurological and unmedicated internal medical conditions, and psychiatric history in first-degree relatives. For exclusion of persons with a present or history of *Diagnostic and Statistical Manual of Mental Disorders, Fourth Edition* (DSM-IV) axis I disorders, we used careful screening *via* phone in the Jena samples and a modified Structured Clinical Interview for DSM-IV Axis I Disorders (SCID-IV) nonpatient version (SCID-NP) interview derived in the Verona/Milano sample. IQ was estimated using the MWT-B (Mehrfach-Wortschatz-Intelligenztest), a German-language inventory (14) similar to the NART (National Adult Reading Text) in the two Jena samples, and the Italian version of the Wechsler Adult Intelligence Scale-Revised (15, 16) for the Verona/Milano sample. This also allowed us to confirm that none of the participants had an estimated IQ of 80 or lower.

For characterization of subclinical depression symptoms, we used the Symptom Checklist 90-Revised (SCL-90-R) scale, as in previous studies including other symptom dimensions. As outlined previously (Besteher et al., *Front Psychiatry*, in press), the SCL-90-R is a self-rating instrument for a broad range of psychopathological symptoms (17). It is very widely used, with more than 1,000 studies making use of it either in clinical measurements of distress/symptoms/psychopathology or for characterization of nonclinical samples (18). Subjects were asked to complete the questionnaire around the time of MRI scanning, indicating their responses to the 90 items on 0–4 Likert-type scales. Previous extensive psychometric studies have established factor analytic solutions, which have also resulted in one “depression” scale, consisting of 13 questions. We used the mean value of these questions to generate the SCL90R score for depression (range 0–4).

An overview of demographic and psychometric data is given in **Table 1**.

**Table 1:** Demographic and psychometric data on study cohorts. Age, gender, IQ, and Symptom Checklist 90-Revised (SCL-90-R) depression subscale values, f—female, m—male. See also Refs. (12, 13).

	Jena_1 (*n* = 177)	Jena_2 (*n* = 141)	Verona/Milano (*n* = 91)
Mean age (SD)	29.8 (ခ±8.93)	32.12 (禒±14.27)	29.13 (禒±7.7)
Age_range	20–60	19–73	18–62
Gender	83 f, 94 m	87 f, 54 m	55 f, 36 m
Mean IQ (SD)	106.23 (ခ±11.5)	115.87 (禒±14.81)	122.39 (禒±8.49)
Mean SCL-90 depression subscale value (SD)	0.36 (ခ±0.44)	0.31 (禒±0. 38)	0.31 (禒±0.25)
SCL-90 depression subscale values (range)	0–2.62	0–2	0–1.23

### Magnetic Resonance Imaging

MRI scans were acquired on a Siemens Tim Trio in Jena and Siemens Magnetom Allegra Syngo MR 2004A for the Verona/Milano sample.

Scan parameters for the three subsamples were as follows:

*Jena-1 sample:* 3 Tesla Siemens Tim Trio scanner (Siemens, Erlangen, Germany), standard quadrature head coil and a T1-weighted axial 3-dimensional magnetization prepared rapid gradient echo (MP-RAGE) sequence (TR 2,300 ms, TE 3.03 ms, α 9°, 192 contiguous sagittal slices, FoV 256 mm, voxel resolution 1 × 1 × 1 mm; acquisition time 5:21 min).

*Jena-2 sample:* 3 Tesla Siemens Prisma fit system (Siemens, Erlangen, Germany), that is, the scanner used for the Jena-1 sample after hardware and software changes to upgrade this to a Prisma system; standard quadrature head coil; MP-RAGE sequence with parameters similar to Jena-1 sample (TR 2,300 ms, TE 2.07 ms, α 9°, 192 contiguous sagittal slices, field-of-view 256 mm, voxel resolution 1 × 1 × 1 mm; acquisition time 5:21 min).

*Verona/Milano sample:* 3 Tesla Magnetom Allegra Syngo MR 2004A (Siemens, Erlangen, Germany); standard head coil for radio frequency transmission and reception of the MRI signal; T1-weighted MP-RAGE sequence (TR 2,060 ms, TE 3.93 ms, α 15°, 160 contiguous sagittal slices, field-of-view 256 mm, voxel resolution 1 × 1 × 1 mm; acquisition time 7:32 min).

Quality assurance for the MRI scans included visual inspection for gross artifacts (after scanning) and the automated quality check implemented in CAT12.

### Voxel-Based Morphometry

We used the CAT 12 toolbox (Computational Anatomy Toolbox 12) of the Structural Brain Mapping group (Christian Gaser, Jena University Hospital, Jena, Germany), a toolbox implemented in SPM12 (Statistical Parametric Mapping, Institute of Neurology, London, UK) for VBM analyses. This included correction for bias-field inhomogeneities; segmentation into gray matter, white matter, and cerebrospinal fluid (19); and spatial normalization with the DARTEL algorithm (20). As described previously, segmentation was further extended by accounting for partial volume effects (21), applying adaptive maximum *a posteriori* estimations (22). We used the automated quality check protocol in CAT12 for further quality assurance. Also, we used smoothing with a Gaussian kernel of 8 mm [full width at half maximum (FWHM)] and an absolute gray matter threshold of 0.1 to minimize incorrect voxel classification, which might result in artifacts or spurious signals.

### Gyrification Analysis

We also used the CAT12 toolbox (C. Gaser, Structural Brain Mapping Group, Jena University Hospital, Jena, Germany) to analyze local gyrification. This analysis is based on an absolute mean curvature approach (23). The cortical surface is extracted, the curvature across the surface is computed, and absolute values are used for further computations. We applied a 15-mm FWHM filter for smoothing.

### Statistics

We used the general linear model implemented in SPM12 for statistical analyses. The analyses included a factor scanner (to correct for the different acquisition sites and scanner settings) and age.

For VBM analyses, we first performed a hippocampal region-of-interest (ROI) analysis (small volume correction) using the AAL toolbox spatial definition of the left and right hippocampi. Given our anatomical hypothesis, we first focused on this area and, using the ROI approach, we restricted the search space (and thus the number of multiple voxel-wise comparisons). This analysis was corrected for multiple comparisons using the cluster-level FWE corrected at *p* < 0.05. In a second step, we provide a whole-brain analysis, first at *p* < 0.05 FWE (cluster-level) corrected levels and then at *p* < 0.001 (uncorrected) for an exploratory view and further hypothesis-generating findings. For all VBM analyses, we included total intracranial volume as a nuisance variable.

For gyrification, we also applied a general linear model and again applied cluster-level FWE corrections at *p* < 0.05.

For all analyses, we computed both positive correlations (anticipating such a relation for compensation/markers of resilience) and negative correlations (conforming to the linear model of a continuum, in this case, across the symptom range within the nonclinical sample).

## Results

**Figure 1:**
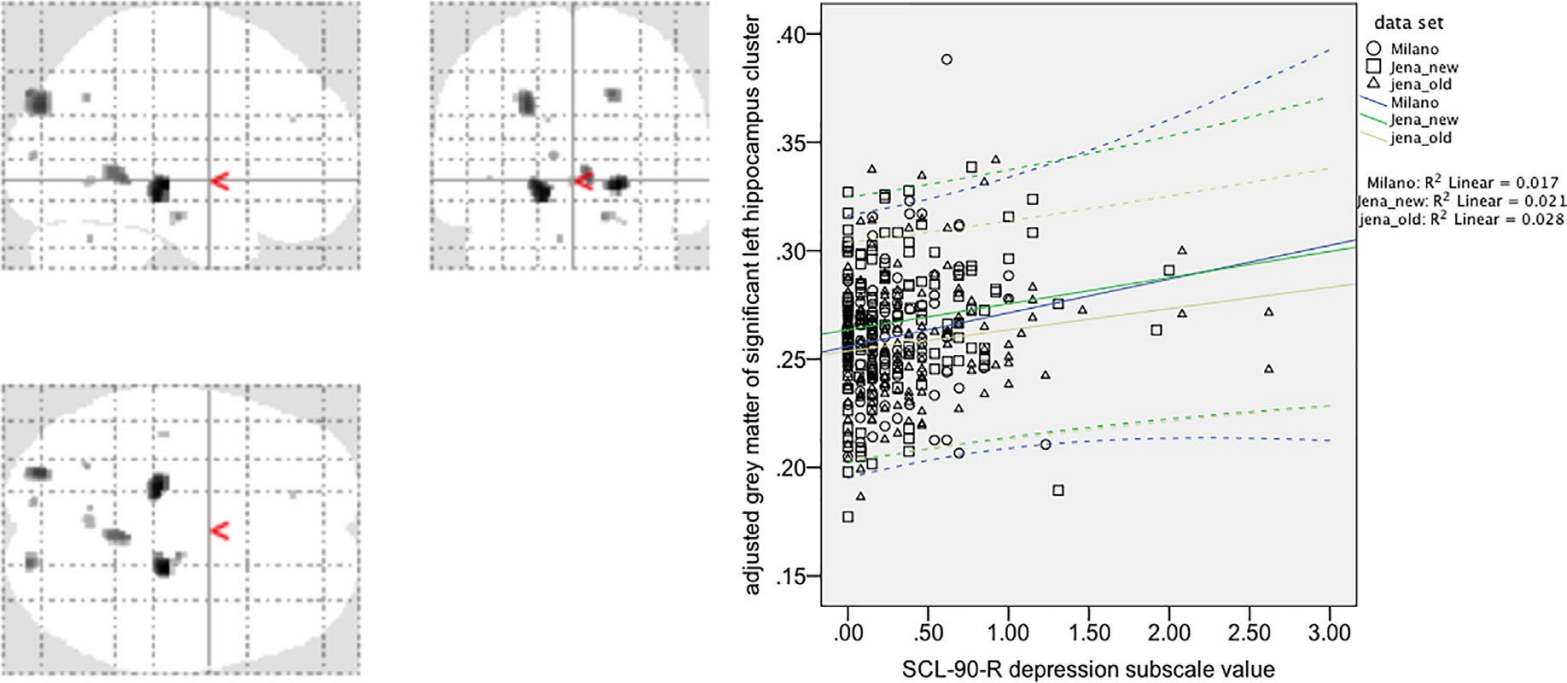
Voxel-based morphometry analysis of positive correlations between gray matter values (GMV) and SCL-90-R depression subscale value in 409 healthy controls (exploratory *post hoc* analysis, *p* < 0.001, uncorrected). Note that the region-of-interest (ROI)/small-volume correction-based analysis for the left hippocampus cluster (maximum coordinate 20; −28; 8; *Z* score 3.59) survived correction for multiple comparisons (*p* = 0.010, FWE corrected at peak level, *p* = 0.042, FWE corrected at cluster level). A correlation plot for the most significant voxel of this left hippocampus cluster [small volume correction (SVC) corrected analysis] is given in the right side of the figure. Spearman´s Rho for the correlation is 0.12 (*p* = 0.015, two-tailed significance at *p* = 0.05), CI 0.024–0.216.

For the hippocampus ROI-based/small-volume correction VBM analysis, we found a significant positive correlation of voxels in a left hippocampus cluster within the ROI search volume (coordinates: −20; −28; 8; *Z* score 3.59; *p* = 0.010, FWE-corrected at peak level, *p* = 0.042, FWE-corrected at cluster level). A similar right hippocampus ROI cluster showed a positive correlation only at uncorrected levels. An overview of the exploratory analysis at *p* < 0.001 peak levels is shown in **Figure 1** and **Table 2** for a comprehensive overview.

**Table 2:** Overview of significant clusters of positive correlations of gray matter values (GMV) with SCL-90-R depression subscale values (based on extended exploratory analysis at *p* < 0.001 uncorrected; for cluster size *k* > 10 voxels).

Anatomical region	Coordinates of peak voxel	*k*	*T*
Right hippocampus	21; −24; 4	77	4.17
*Left hippocampus	−16; −27; −9	131	4.1
Left superior/middle occipital gyrus	−24; −84; 33	132	3.79
Right cerebellum (/vermis)	6; −45; −2	62	3.62
Right cuneus/right sup. occipital cortex	18; −90; 38	39	3.58
Right parahippocampal cortex	14; –16; –20	17	3.43
Left cerebellum (/vermis)	−2; −62; −3	14	3.27

*indicates clusters surviving correction for multiple comparisons (p < 0.05, FWE-corrected).

We did not find negative correlations of hippocampal ROI/small-volume-based analyses, and exploratory analysis (*p* < 0.001, uncorrected) only revealed a small 5-voxel cluster in the right middle frontal cortex.

**Figure 2:**
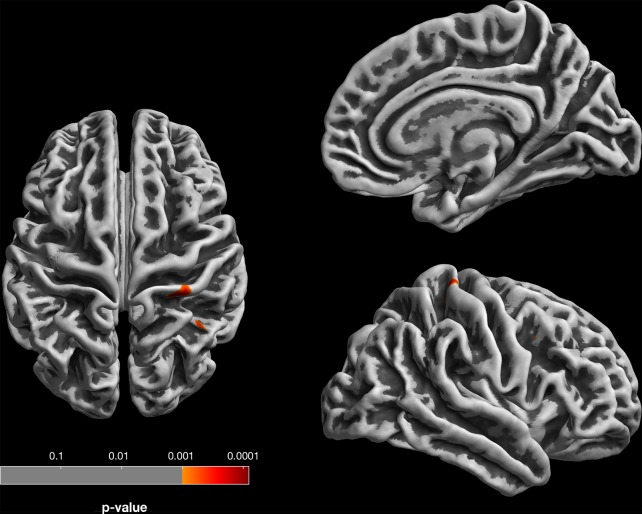
Gyrification analysis (based on absolute mean curvature approach; 23) of positive correlations between local gyrification and SCL-90-R depression subscale. For visualization, results are displayed at *p* < 0.001, uncorrected, thresholds; the right postcentral gyrus finding survives *p* < 0.05 FWE correction at cluster level.

Gyrification analysis of correlation with subclinical depressive symptoms in the neocortex (at corrected levels) showed only one positive correlation in a right postcentral cortical cluster (*p* = 0.031, FWE-corrected at cluster level) and no negative correlations (**Figure 2**).

## Discussion

Subclinical depressive symptoms, defined as minor affective self-report features, are associated with brain gray matter variation in a part of the hippocampus. This main finding of our study expands the current understanding of dimensional psychopathology to biological findings and also provides some potential clues to brain structural markers of resilience. The positive correlation of subclinical symptom load with hippocampal volume prompts our discussion to effects of resilience, which might distinguish nonclinical from clinical subjects.

Our findings add to a growing recent literature expanding the identification of correlations between depressive symptoms and brain structure into the subclinical range. Initial studies, mostly in smaller samples, failed to identify a correlation with hippocampal volume but suggested prefrontal areas to be involved in subclinical depression (24, 25). Similarly, subsequent smaller studies have shown mixed effects hinting to associations with anterior cingulate cortex volume, pronounced in female subjects, but not in hippocampal volume (26, 27). Larger studies were then conducted in elderly community subjects subsequently, but these have again been inconsistent, especially regarding hippocampal volume (28, 29). The different age ranges of these previous studies make it difficult to compare these findings, especially because interaction effects with age are possible and likely. Two most recent studies in middle-aged cohorts (*n* = 192 and *n* = 80), however, did find some association of (negative) symptoms with hippocampal texture and volume (30, 31).

Although these previous studies give some indication of hippocampal volume being related to depressive symptoms in nonclinical subjects, most findings are limited to either small samples or particular age ranges. Our hippocampal finding therefore establishes the association in a large multicenter study across three cohorts across a wide age range.

The interpretation of these findings needs to consider the positive direction of correlations. In clinical studies of manifest depression, reduced hippocampal volume is related to acute episodes (9). Several interpretations of our findings are therefore at hand. First, the positive correlation could signal a resilience effect. Although showing slightly higher depression scores, healthy subjects with larger hippocampal volume might display compensation effects that prevent them from transitioning to higher levels of depressive symptoms. So far, it is unclear which cellular effects might be associated with such resilience to expressing a disease phenotype. A most recent study has implicated volume reduction of subregions CA2–CA4 of the hippocampus to be associated with first onset of clinical depression (whereas reductions in CA1 and the dentate gyrus were linked to recurrent disease) (32). Clearly, our cross-sectional design limits prediction of whether subsequent depression is more likely in those subjects with larger hippocampi (and higher resilience) or those with smaller hippocampi. An alternative possible interpretation would be that other factors, such as genetic liability, determine hippocampal volume to an extent that might result in such a positive correlation. Neither the previous studies nor our own considered indicators like polygenic risk scores to further explore this possibility.

We have noted in previous studies that subclinical or minute symptoms in nonclinical subjects might show positive rather than negative correlations, thus setting them apart from studies in clinical populations (12, 13). Future studies might therefore have to consider nonlinear effects across larger symptom ranges; for example, when including both clinical and nonclinical subjects in association analyses, the direction of correlations might be different across such putative disease-related spectra. This interpretation also provides a mechanistic account of the failure of previous studies aiming to identify hippocampal volume reduction as a precursor of incipient depression in elderly subjects (33), as multiple factors including genetic load and stress, but also resilience might interact to produce dynamic changes in hippocampal volume.

In addition to our VBM findings, our gyrification analysis (including neocortical but not hippocampal surfaces) did not show an association with minor depressive features other than a right postcentral cortical cluster. Although this is consistent with the notion that this marker of early neurodevelopment is not related to temporary fluctuations, it does not argue for early neocortical abnormalities related to subclinical symptom expression.

Our study needs to consider some methodological limitations. Although larger than most other studies on brain structure in subclinical symptoms and including multicenter data, further replication of our positive association seems warranted. Also, larger samples across the life span might be used to identify changes in associations in particular (adult) age ranges or linked to other demographic factors. The cross-sectional nature of our data (and thus lack of follow-up) precluded the use of prediction models to be tested.

Minor or subclinical depressive symptoms are increasingly recognized to be of relevance for our understanding of major depression and early intervention, even if those symptoms are only transient in nature and often unspecific reactions to stress or negative life events. There is increasing evidence from multiple studies suggesting that psychotherapy for subclinical depression not only is effective but also might reduce the incidence of major depression (34, 35). Hippocampal volume as a putative biomarker might aid in this endeavor (3), but compensation and resilience have not adequately been considered in these previous models. Our findings argue for an integration of anticorrelations as markers of resilience to be taken into account in mechanistic models relating brain structure to depressive symptoms across a wide range of phenotypic expression, including the subclinical range.

## Ethics Statement

The authors assert that all procedures contributing to this work comply with the ethical standards of the relevant national and institutional committees on human experimentation and with the Helsinki Declaration of 1975, as revised in 2008. All study participants provided written informed consent to protocols approved by the local Ethics Committee of Jena University Medical School, and the Ethics Committee of the Azienda Ospedaliera Universitaria of Verona, respectively.

## Author Contributions

BB, PB, and IN conceived of the study. BB, RS, PB, MB, and IN contributed to recruitment. BB, LS, CG, and IN contributed to data analysis. BB and IN wrote the first draft of the manuscript. All authors contributed to manuscript revision and approval.

## Funding

The study was in part supported by grants of IZKF Jena to BB (Jena-2 sample) as well as of the Italian Ministry of Health to MB (GR-2010-2319022), and to PB and LS (RF-2016-02364582).

## Conflict of Interest Statement

The authors declare that the research was conducted in the absence of any commercial or financial relationships that could be construed as a potential conflict of interest.
